# Rapid Response to the Combination of Lenvatinib and Sintilimab in a Pancreatic Acinar Cell Carcinoma Patient With Elevated Alpha-Fetoprotein: A Case Report

**DOI:** 10.3389/fonc.2021.692480

**Published:** 2021-10-20

**Authors:** Lanqun Qin, Jie Shen, Yueling Yang, Zhengyun Zou

**Affiliations:** ^1^ The Comprehensive Cancer Centre of Nanjing Drum Tower Hospital, Clinical College of Nanjing Medical University, Nanjing, China; ^2^ The Comprehensive Cancer Centre of Nanjing Drum Tower Hospital, Medical School of Nanjing University & Clinical Cancer Institute of Nanjing University, Nanjing, China; ^3^ The Comprehensive Cancer Centre of Nanjing Drum Tower Hospital, Clinical College of Nanjing University of Chinese Medicine, Nanjing, China

**Keywords:** alpha-fetoprotein, sintilimab, lenvatinib, pancreatic acinar cell carcinoma, case report

## Abstract

A 48-year old woman was diagnosed with metastatic pancreatic acinar cell carcinoma (PACC) and with a marked elevation in alpha-fetoprotein (AFP), this being a recognized but uncommon feature of PACC. As she refused chemotherapy, the combined therapy of lenvatinib and sintilimab (lenvatinib 8 mg, orally, qd; and sintilimab 100 mg, intravenous glucose tolerance test, q21d) was given, which conferred significant tumor shrinkage and long progression-free survival (>21 months). This study is the first report and description of a PACC demonstrating favorable response to the combination therapy of an antiangiogenic agent and immunotherapy.

## Introduction

Acinar cell carcinoma (ACC) is an epithelial tumor similar to the acinar cells of the exocrine gland (1). It can originate from the pancreas, salivary gland, prostate, and lung (2), among which pancreatic acinar cell carcinoma (PACC) is the most common. PACC, which was first reported in 1908, accounts for 1%–2% of all pancreatic tumors and mainly occurs in the pancreas head of medium-elderly men (3, 4). However, alpha-fetoprotein (AFP)-elevated PACC is rare with no more than 30 cases being reported worldwide (5, 6).

Since PACC is generally asymptomatic in the early stages, clinical presentation is often delayed, and about 50% of cases already have metastatic disease at diagnosis. The liver is the most commonly affected site (7). There are no specific tumor indicators, but elevated AFP has been reported (7, 8), which can level up to >160,780 ng/ml (9). This may be related to the fact that AFP is a secreted product of liver cancer cells.

## Case Presentation

**Figure 1:**
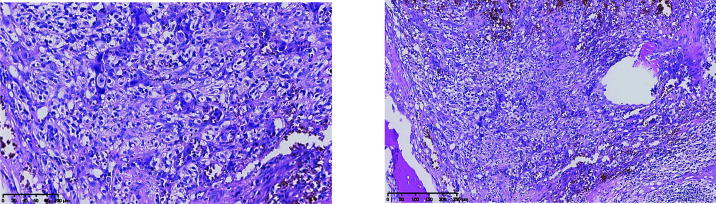
Pathology after hip replacement (H&E staining) showed heterogeneous epithelial cell infiltrating between trabeculae. The left was taken at ×20, and the right was taken at ×10.

**Figure 2:**
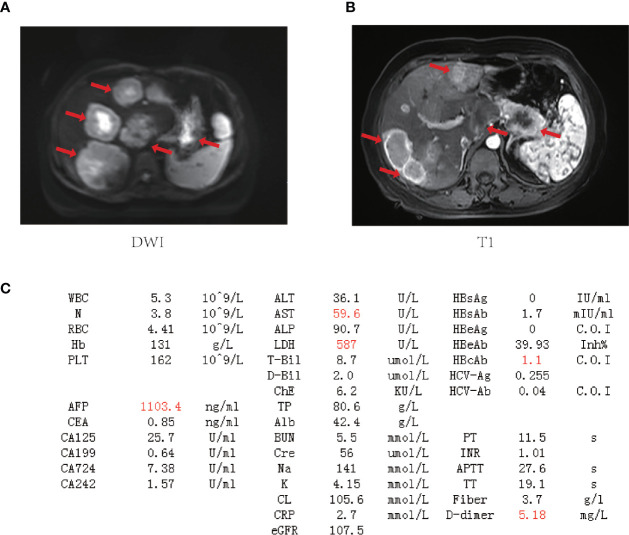
The baseline laboratory and imaging examinations for the patient. **(A, B)** Magnetic resonance imaging (MRI) of the upper abdomen. Tumor lesions are marked by red arrows. **(C)** Laboratory data on admission (outliers are highlighted in red). WBC, white blood cell count; N, neutrophil count; RBC, red blood cell count; Hb, hemoglobin; PLT, blood platelet count; AFP, alpha-fetoprotein; CEA, carcinoembryonic antigen; CA125, carbohydrate antigen 125; CA199, carbohydrate antigen 19-9; CA724, carbohydrate antigen 724; CA242, carbohydrate antigen 242; ALT, alanine aminotransferase; AST, aspartate aminotransferase; ALP, alkaline phosphatase; LDH, lactate dehydrogenase; T-Bil, total bilirubin; D-Bil, direct bilirubin; ChE, cholinesterase; TP, total protein; Alb, albumin; BUN, blood urea nitrogen; Cre, creatinine; Na, sodium; K, potassium; CL, chlorine; CRP, C-reactive protein; eGFR, glomerular filtration rate; HBsAg, hepatitis B surface antigen; HBsAb, hepatitis B surface antibody; HBeAg, hepatitis B e antigen; HBeAb, hepatitis B e antibody; HBcAg, hepatitis B core antigen; HBcAb, hepatitis B core antibody; HCV-Ag, hepatitis C virus antigen; HCV-Ab, hepatitis C virus antibody; PT, prothrombin time; INR, international normalized ratio; APTT, activated partial thromboplastin time; TT, thrombin time.

A 48-year-old female underwent right total hip replacement because of right hip trauma on December 4, 2019. Pathological findings showed that local tissue of the femoral head was necrotic with a heterogeneous epithelial infiltration between the trabeculae, consistent with a metastatic adenocarcinoma (**Figure 1**). Immunohistochemistry was negative for markers CD7, CK20, TTF1, P40, PAX8, S100, HMB45, Hept1, GATA3, SALL4, HCG, GPC3, or NY-ESO-1 but positive for CK and Villin. However, the primary tumor still remained unclear, as she refused liver biopsy. Abdominal MRI showed multiple occupations in the liver and around the pancreatic tail (**Figure 2**), while gynecological B-ultrasound and gastroenteroscopy revealed no abnormality. The patient had no history of hepatitis but had diabetes.

Despite the elevated AFP, the lack of rapid filling and rapid washout of contrast in the liver lesions in CT and absence of antecedents such as hepatitis or alcohol consumption goes against the liver lesions being hepatocellular carcinoma (HCC). After multidisciplinary discussion and literature retrieval, the patient was eventually diagnosed with PACC clinically based on her radiographic data: large diameter, common internal necrosis, exophytic growth, and a well-defined margin with enhanced capsule, often invading peripheral blood vessels, which are the typical radiographic features of PACC (10).

**Figure 3:**
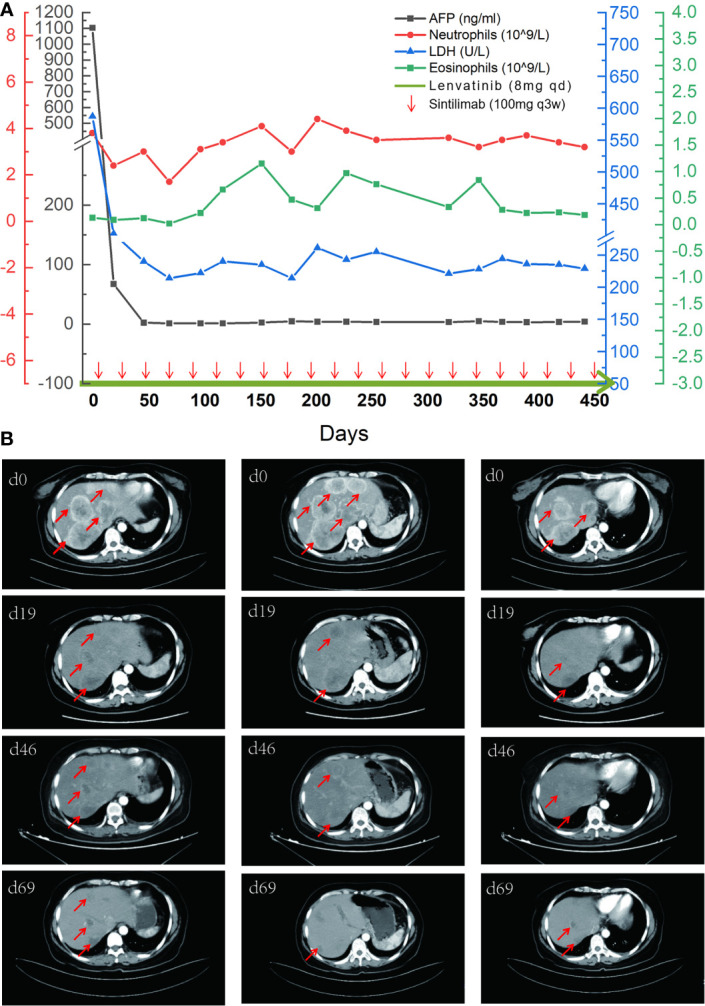
The comparison of tumor indicators and CT images. **(A)** A timeline outlining the treatment course (lenvatinib 8 mg, orally, qd; and sintilimab 100 mg, intravenous glucose tolerance test, q21d) and changes of laboratory indicators including alpha-fetoprotein (AFP), neutrophils, lactate dehydrogenase (LDH), and eosinophils. **(B)** Imaging changes of the first four times of evaluation with tumor lesions marked by red arrows.

There is no standard treatment for PACC due to its rarity, and the patient refused chemotherapy for fear of chemotherapy-related side effects. Abdominal CT showed multiple masses in the liver and a solitary 3.7-cm mass in the pancreatic tail with a rich blood supply to the tumor, which is the key point to choose antiangiogenic agents. And through literature review, we also found synergy effects of antiangiogenic agents combined with anti-PD-1 antibody. After discussion, the patient consented to undergo a combination therapy of sintilimab (a multitargeted tyrosine kinase inhibitor (TKI)) and lenvatinib (an analog of pembrolizumab with a much lower cost). Three weeks into the treatment, her AFP levels (**Figure 3A**) dropped dramatically, and tumor lesions (**Figure 3B**) shrank significantly. The patient has demonstrated a partial response (PR) of 21 months since the start of treatment. No severe grade 3 and beyond side effects were observed.

## Discussion

Due to its rarity, there is no standard treatment for PACC. Complete resection is always the preferred option (11), and chemotherapy often refers to the regimens of colorectal cancer or ductal adenocarcinoma based on their similar genetic changes for those who cannot undergo resection (12). A retrospective study showed that about 50%–60% patients diagnosed with metastatic PACC responded to a possible combination of gemcitabine, docetaxel, and erlotinib (13). Antoine et al. also reported a PACC case with liver metastasis who survived for 37 months through a combined therapy with gemcitabine, irinotecan, docetaxel, etc (14). Some studies evaluated the chemical sensitivity of drugs by ATP-CRA and found that cyclophosphamide is the most ideal drug for AFP-producing PACC (15).

The combination therapy of antiangiogenic agents and immunotherapy has shown certain efficacy in a variety of tumors, such as renal cell carcinoma (16, 17), HCC (18), and malignant melanoma (19). Recently, this combination also showed a strong therapeutic effect for a tumor mutational burden (TMB)-high pancreatic cancer patient after a series of ineffective treatments (20). Unfortunately, the patient refused to undergo liver biopsy, so no specimen was available for further analysis of mismatch repair deficient (dMMR), PD-L1, TMB, and other immune-related markers.

The liver is an organ with high vascularization and immunogenicity, and liver metastasis often suggests poor prognosis. Lenvatinib, a multitargeted TKI, definitely had a certain effect because of the rich blood supply to the tumor region. Meanwhile, TKIs could also induce vascular normalization and thus reduce hypoxia, increase intratumoral infiltration of cytotoxic T lymphocytes, and decrease regulatory T-lymphocyte recruitment, resulting in a more favorable immune microenvironment to enhance the antitumor activity of immunotherapy (21). Another recent case report found that CD8+ T cells may respond rapidly to pembrolizumab after exposure to lenvatinib, signifying that the CD8+ T cell’s sensitivity to immunotherapy was improved in the short term after treatment with TKIs (22). In all, antiangiogenic agents combined with immunotherapy can produce synergy effects.

Many indicators were reported to be used to predict the curative effect of immunotherapy, such as lactate dehydrogenase (LDH) (23), neutrophil-to-lymphocyte ratio (20), and neutrophil count (24, 25). But these indicators showed no relevance to the curative effect in our case. As for the patient, the baseline of eosinophil level was in the normal range and gradually increased during treatment (**Figure 3A**), suggesting that eosinophil may play an active role in the immune response. AFP may also be used as an indicator of response to the treatment because it was elevated before the treatment.

In summary, to the best of our knowledge, this is the first report of favorable response to the combination of antiangiogenic agents and immunotherapy in a PACC patient, which provides a new therapeutic approach for many tumors that have reached the treatment bottleneck or rare tumors that have no standard treatment.

## Data Availability Statement

The original contributions presented in the study are included in the article/supplementary material. Further inquiries can be directed to the corresponding author.

## Ethics Statement

Written informed consent was obtained from the individual(s) for the publication of any potentially identifiable images or data included in this article.

## Author Contributions

LQ and JS contributed equally. All authors contributed to the article and approved the submitted version.

## Funding

This study was supported by the National Natural Science Foundation of China (Nos. 81872484 and 82073365).

## Conflict of Interest

The authors declare that the research was conducted in the absence of any commercial or financial relationships that could be construed as a potential conflict of interest.

## Publisher’s Note

All claims expressed in this article are solely those of the authors and do not necessarily represent those of their affiliated organizations, or those of the publisher, the editors and the reviewers. Any product that may be evaluated in this article, or claim that may be made by its manufacturer, is not guaranteed or endorsed by the publisher.
